# Cost-effectiveness analysis of cabozantinib plus atezolizumab for advanced hepatocellular carcinoma

**DOI:** 10.3389/fphar.2025.1556304

**Published:** 2025-10-09

**Authors:** Yan Zhu, Cheng He, Shi-Chao Cao, Hui-Jun Li, Ru-Xue Cai, Zhao-Jun Bai, Tong-Xia Xia

**Affiliations:** 1 Department of Pharmacy, Affiliated Hospital of Zunyi Medical University, Zunyi, Guizhou, China; 2 Department of Pharmacy, Zunyi Bozhou District People’s Hospital, Zunyi, Guizhou, China; 3 Sinopharm Group Tongjitang (Guizhou) Pharmaceutical Co., Ltd., Economic and Technological Development District, Guiyang, Guizhou, China; 4 Guangxi Shenli Pharmaceutical Co., Ltd., Yulin, Guangxi, China; 5 School of Nursing, Zunyi Medical University, Zunyi, Guizhou, China

**Keywords:** cost-effectiveness analysis, advanced hepatocellular carcinoma, cabozantinib, atezolizumab, sorafenib

## Abstract

**Background:**

Cabozantinib combined with atezolizumab has been shown to prolong progression-free survival in first-line treatment of advanced hepatocellular carcinoma (HCC). However, the cost-effectiveness of this regimen remains unknown. This study aimed to evaluate the cost-effectiveness of cabozantinib plus atezolizumab compared with sorafenib for first-line treatment of advanced HCC from the perspectives of the Chinese health system and the US payers.

**Methods:**

A partitioned survival model was constructed based on a phase III randomized clinical trial (COSMIC-312) to compare the health benefits and economic outcomes of cabozantinib plus atezolizumab versus sorafenib for the treatment of hepatocellular carcinoma. Costs and utilities were obtained from published literature. Data recorded included quality-adjusted life years (QALYs), life years (LYs), and incremental cost-effectiveness ratio (ICER). One-way and probabilistic sensitivity analyses were conducted to test the robustness of the results. Subgroup analyses were also performed.

**Results:**

From the perspectives of the US payers and the Chinese health system, the base-case ICER values for cabozantinib plus atezolizumab compared to sorafenib were $-2,731,994.74/QALY and $-2,225,520.14/QALY, respectively. Sorafenib achieved an absolute dominance in terms of cost-effectiveness, offering greater benefits at a lower cost. The models were most sensitive to the utility values for progression-free survival and overall survival. Subgroup analyses also demonstrated that cabozantinib plus atezolizumab was unlikely to be cost-effective as a first-line treatment for advanced HCC.

**Conclusion:**

Cabozantinib plus atezolizumab was not a cost-effective treatment option for HCC when compared to sorafenib from both the Chinese healthcare system and the US payer perspectives.

## 1 Introduction

Hepatocellular carcinoma (HCC) ranks as the sixth most frequently diagnosed cancer and stands as the third leading cause of cancer-related mortality on a global scale (Bray et al., 2024). HCC and intrahepatic cholangiocarcinoma (iCCA) constitute the two major histological subtypes of primary liver cancer (Rumgay et al., 2022). HCC accounts for approximately 80% of all liver cancer cases worldwide, with an age-standardized incidence rate (ASR) of 7.3 per 100,000 person-years, making it the predominant pathological type of primary liver cancer (Rumgay et al., 2022). The prognosis of HCC depends on the tumor stage, the degree of underlying liver dysfunction, and the patient’s performance status (Forner et al., 2018). While early-stage HCC may be addressed with local therapeutic interventions such as surgical resection, radiotherapy, and transarterial chemoembolization (TACE), the majority of patients are diagnosed at an advanced stage, rendering them ineligible for surgical treatment. Consequently, systemic therapy emerges as a vital treatment approach for these individuals (Finn et al., 2020).

It is estimated that approximately 50%–60% of HCC patients will receive systemic therapy. Currently developed systemic treatments include tyrosine kinase inhibitors (TKIs), immune checkpoint inhibitors (ICIs), and anti-vascular endothelial growth factor (VEGF) antibodies (Llovet et al., 2021). Despite the notable progress in systemic therapies over recent years, the prognosis for the majority of patients with HCC remains grim, highlighting the urgent need for additional effective treatment options to serve a broader patient population (Yang et al., 2024). Sorafenib has been shown to significantly improve overall survival (OS) and has been the gold standard for the systemic treatment of advanced HCC since its approval in 2007 (Llovet et al., 2008). However, sorafenib resistance develops in most patients, limiting its clinical benefits (Gauthier and Ho, 2013).

Studies on treatment regimens incorporating ICIs are currently a focus in HCC nowadays, especially in combination with TKI drugs (Zheng et al., 2025). Cabozantinib is a multi-targeted TKI that exhibits significant activity in inhibiting HCC tumor cells. In the CELESTIAL trial, cabozantinib demonstrated a significant improvement in median overall survival and progression-free survival (PFS) compared to placebo in patients who had failed sorafenib therapy (Abou-Alfa et al., 2018). The activity of cabozantinib may be attributed to its dual inhibition of VEGFR2 and MET signaling pathways. Given that one of the mechanisms of sorafenib resistance is the overexpression of MET in HCC cells (Wu et al., 2024; Xiang et al., 2014), the additional inhibitory effects of cabozantinib on MET make it a preferred second-line treatment option for patients who have developed resistance to sorafenib.

The potential of combination therapies has been underscored by the positive outcomes of the IMbrave150 and HIMALAYA trials, which demonstrated promising activity and safety profiles (Finn et al., 2020; Sangro et al., 2024). The COSMIC-312 trial further highlighted the clinical benefits of combining cabozantinib, a TKI, with the immune checkpoint inhibitor atezolizumab in the treatment of solid tumors, including advanced HCC. This trial evaluated the efficacy of cabozantinib plus atezolizumab compared with sorafenib as first-line systemic therapy for advanced hepatocellular carcinoma. The study reported that the combination therapy significantly extended PFS compared to sorafenib (6.9 months vs. 4.3 months; hazard ratio [HR], 0.74; 99% confidence interval [CI], 0.56–0.97), but there was no significant difference in OS between the two treatment groups (16.5 months vs. 15.5 months; HR, 0.98; 96% CI, 0.78–1.24) (Yau et al., 2024). Although no significant differences in overall survival rates were observed, the benefits of the combination therapy regimen on progression-free survival and disease control still noteworthy, may become a new treatment option. In terms of cost, the combination therapy is expensive, and its economic assessment in regions with different economic conditions remains unclear. As a developed country, the United States spends 20% of its gross domestic product on healthcare (Alspaugh et al., 2021). The high medical costs will bring enormous financial pressure to the healthcare system. After the implementation of the Affordable Care Act, the Budget Control Act, and other policies, the ability of some patients to afford medical services has been somewhat improved, but for many others, it is still far from sufficient. (Jones and Kantarjian, 2019; Zheng and Sandhu, 2023; Mullangi and Eagle, 2023). As the largest developing country, China has achieved near-universal health insurance coverage, which benefits a large number of people, but this also means increased financial pressure (Chen et al., 2022; Yip et al., 2019). Decisions on healthcare policy and resource allocation will significantly affect the overall efficiency and effectiveness of the healthcare system. Therefore, this study aims to construct a partitioned survival model based on the data from the COSMIC-312 trial to compare the cost-effectiveness of cabozantinib plus atezolizumab versus sorafenib as first-line treatment for advanced, unresectable hepatocellular carcinoma from the perspectives of the Chinese healthcare system and the US payers, providing a reference for clinical drug selection decisions.

## 2 Methods

### 2.1 Population and interventions

The target patient population for this analysis was assumed to be similar to that of the COSMIC-312 trial, consisting of patients aged 18 years or older with histologically confirmed, previously untreated advanced HCC, Barcelona Clinic Liver Cancer (BCLC) stage B or C, Child-Pugh class A, and Eastern Cooperative Oncology Group (ECOG) performance status of 0 or 1. Two treatment regimens for advanced HCC were compared: cabozantinib plus atezolizumab combination (cabozantinib 40 mg orally once daily plus atezolizumab 1200 mg intravenously every 3 weeks) versus sorafenib monotherapy (sorafenib 400 mg orally twice daily) as first-line therapy. Treatment was discontinued upon disease progression or intolerable adverse events, and patients then received best supportive care (BSC) and subsequent treatments until death. All deceased patients were considered to receive end-of-life care.

### 2.2 Model overview

**FIGURE 1:**
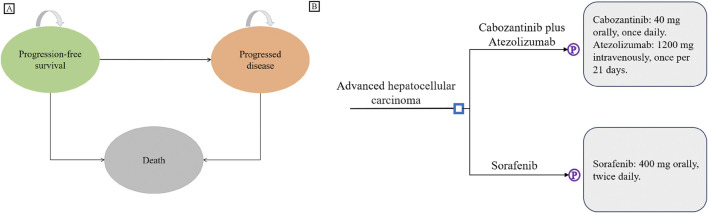
Model Structure. **(A)** Partitioned survival model overview. **(B)** Interventions for advanced hepatocellular carcinoma in first-line treatment.

This study reports on the basis of the Consolidated Health Economic Evaluation Reporting Standards 2022 (CHEERS 2022) checklist (Husereau, et al., 2022), which is included as Supplementary Table S1 within the Supplementary Materials. A partitioned survival was constructed using Excel for cost-effectiveness analysis (as depicted in Figure 1). Patients were assumed to be in the PFS state at baseline, and could progress to death from any state. Time horizon was set to 5 years, with a cycle length of 3 weeks. By the end of the final cycle, the cumulative mortality rate was projected to exceed 95%. The study was conducted from the dual perspectives of the US payers and the Chinese healthcare system. The primary outcomes measured in the analysis were costs, life-years (LYs), quality-adjusted life-years (QALYs), and incremental cost-effectiveness ratio (ICER). Willingness-to-pay (WTP) thresholds were set at $150,000/QALY and $37,863/QALY for the US and China, respectively (Neumann et al., 2014; Liu et al. 2020). A treatment strategy was deemed not cost-effective if its ICER surpassed these respective WTP thresholds. Annual discount rates for both costs and utilities were set at the commonly used rate of 5% (Attema et al., 2018).

### 2.3 Survival estimate

Given the limited follow-up time in the COSMIC-312 trial, extrapolation of the reported survival curves is necessary to obtain the required survival data. Using algorithms developed by Guyot and Wei (Guyot et al., 2012; Wei and Royston, 2017), pseudo-patient individual data was generated using Stata 17 (StataCorp LLC. College Station, Texas), and the survival curve was reconstructed and extended. The individual patient data were obtained from published clinical trials by digitizing the Kaplan-Meier survival curves using GetData Graph Digitizer version 2.25. Parametric survival models were fitted to these data using various distributions, including Weibull, Exponential, Gompertz, Log-logistic, and Log-normal. To reflect the most realistic clinical outcomes and physiological course, the distributions with the lowest Akaike information criterion (AIC) and Bayesian information criterion (BIC) values were not exclusively used in this study. Instead, both the Weibull and Log-normal distributions were fitted to the OS and PFS curves for both treatment arms. The AIC and BIC values of different Kaplan–Meier curve fitting distributions, as well as the goodness of fit, are presented in the Supplementary Table S2; Supplementary Figure S1. The distribution parameters of the survival curves are shown in Table 1.

**TABLE 1:** Key clinical parameters and health utility inputs.

Parameter	Cabozantinib plus atezolizumab	Sorafenib	Distribution	References
Weibull survival model for OS	scale = 0.016686 shape = 1.319320	scale = 0.032411 shape = 1.079312		Yau et al. (2024)
Log-normal survival model for PFS	μ = 1.911015 σ = 1.086205	μ = 1.615714 σ = 1.113573		Yau et al. (2024)
Incidence of grade 3/4 AEs				
PPES	0.08	0.08	Beta (88.277, 1015.183)	Kelley et al. (2022)
AST increased	0.09	0.035	Beta (87.306, 882.765) (92.644, 2554.316)	Kelley et al. (2022)
ALT increased	0.09	0.025	Beta (87.306, 882.765) (93.614, 3650.946)	Kelley et al. (2022)
Hypertension	0.09	0.08	Beta (87.306, 882.765) (88.277, 1015.183)	Kelley et al. (2022)
Fatigue	0.03	0.04	Beta (93.129, 3011.165) (92.158, 2211.802)	Kelley et al. (2022)
Utilities				
PFS	0.76 (0.61–0.91)		Beta (22.908, 7.234)	Su et al. (2021)
PD	0.68 (0.54–0.82)		Beta (28.322, 13.328)	Su et al. (2021)
Disutility due to AEs				
PPES	−0.116		Beta (84.783, 646.108)	Parikh et al. (2017)
AST increased	0		Beta	Xu et al. (2019)
ALT increased	0		Beta	Xu et al. (2019)
Hypertension	−0.012		Beta (94.876, 7811.418)	Meng et al. (2022)
Fatigue	−0.11		Beta (85.366, 690.685)	Chiang et al. (2021)
Proportion receiving subsequent treatment	0.2	0.37	Beta (76.632, 306.528) (60.135, 102.392)	Kelley et al. (2022)

AEs, adverse events; ALT, alanine aminotransferase; AST, aspartate aminotransferase; OS, overall survival; PD, progression disease; PFS, progression-free survival; PPES, Palmar-plantar erythrodysaesthesia syndrome.

### 2.4 Cost and utility

For this analysis, only direct medical costs were considered. These costs primarily included: drug costs, administration fees, follow-up care, imaging costs, best supportive care costs, treatment costs for serious adverse events, and costs of subsequent and end-of-life care. To facilitate comparison, all costs for both countries were reported in the US dollars. The exchange rate between the United States dollar (USD) and the Chinese Yuan (CNY) in 2024 was as follows: 1 USD = 7.23 CNY (State Administration of Foreign Exchange, 2024).

Drug prices in this study were sourced from online databases (Menet, 2024; Drugshk, 2024; Medicare Part B, 2024; Medicare Part D, 2024). Other costs were obtained from published literature (Su et al., 2021; Parikh et al., 2017; Xu et al., 2019; Meng et al., 2022; Wu et al., 2018; Lu et al., 2017; Sun et al., 2022; Chen et al., 2023; Shi et al., 2018; Qin et al., 2018; Kobayashi et al., 2019; Soto-Perez-De-Celis et al., 2019; Li et al., 2022; Zhan et al., 2021; Sieg et al., 2020). As cabozantinib is not yet available in mainland China, we referenced prices from Hong Kong. Serious adverse events (AEs) were defined as grade 3 or 4 adverse reactions. The incidence of AEs was derived from the COSMIC-312 study (Kelley et al., 2022), including hypertension, increased aspartate aminotransferase/alanine aminotransferase, fatigue, and hand-foot syndrome. Costs associated with AEs were calculated by multiplying the probability of experiencing an AE by the cost per event. And we assume that all AEs occur in the first cycle and only happen once. After disease progression, 20% of patients in the combination therapy group and 37% of patients in the sorafenib group received subsequent systemic therapy. All costs are shown in Table 2.

**TABLE 2:** Cost inputs.

Parameter	China mean (range)	US mean (range)	Distribution
Cost ($)			
Cabozantinib per 40 mg tablet	299.67 (239.74–359.60) (Drugshk, 2024)	898.34 (718.67–1078.01) (Medicare Part D, 2024)	Gamma (96.053, 3.120) (96.038, 9.354)
Atezolizumab per 1200 mg	4574.38 (3659.50–5489.26) (Menet, 2024)	11,177.52 (8942.02–13413.02) (Medicare Part B, 2024)	Gamma (95.997, 47.641) (96.083, 116.332)
Sorafenib per 200 mg	12.35 (9.88–14.82) (Menet, 2024)	179.23 (143.38–215.08) (Medicare Part D, 2024)	Gamma (96.040, 0.129) (96.019, 1.867)
Drug administration	41.00 (32.80–49.2) (Wu, et al., 2018)	147.44 (117.95–176.93) (Zhang, et al., 2021)	Gamma (96.040, 0.427) (96.027, 1.535)
Follow-up	39.66 (31.73–47.59) (Chen et al., 2023)	212.00 (169.60–254.40) (Li et al., 2022)	Gamma (96.088, 0.413) (96.040, 2.207)
CT imaging (per 6 weeks)	85.01 (68.01–102.01) (Sun et al., 2022)	783.00 (626.40–939.60) (Zhang et al., 2021)	Gamma (96.063, 0.885) (96.040, 8.153)
Best support care	337.50 (270.00–405.00) (Lu et al., 2017)	2871.00 (2296.80–3445.20) (Su et al., 2021)	Gamma (96.040, 3.514) (96.040, 29.894)
End-of-life care	278.21 (222.57–333.85) (Sun et al., 2022)	7739.00 (6191.20–9286.80) (Soto-Perez-De-Celis et al., 2019)	Gamma (96.047, 2.897) (96.040, 80.581)
Subsequent treatment			
Cabozantinib plus Atezolizumab	394.73 (315.78–473.67) (Menet, 2024; Kelley et al., 2022)	5712.40 (4569.92–6854.88) (Medicare Part B, 2024; Medicare Part D, 2024; Kelley et al., 2022)	Gamma (96.042, 4.110) (96.040, 59.479)
Sorafenib	1355.79 (1084.63–1626.95) (Menet, 2024; Kelley et al., 2022)	12,715.35 (10,172.28–15258.42) (Medicare Part B, 2024; Medicare Part D, 2024; Kelley et al., 2022)	Gamma (96.039, 14.117) (96.040, 132.396)
Cost of managing AEs (grade 3/4) per event			
PPES	12.00 (9.60–14.40) (Shi et al., 2018)	385.00 (308.00–462.00) (Sieg et al., 2020)	Gamma (96.040, 0.125) (96.040, 4.009)
AST/ALT increased	59.00 (47.2–70.8) (Qin et al., 2018)	59.00 (47.20–70.80) (Qin et al., 2018)	Gamma (96.040, 0.614)
Hypertension	37.00 (29.6–44.4) (Qin, et al., 2018)	78.00 (62.40–93.60) (Sieg et al., 2020)	Gamma (96.040, 0.385) (96.040, 0.812)
Fatigue	3.00 (2.4–3.6) (Zhou et al., 2022)	93.00 (74.40–111.60) (Chiang et al., 2021)	Gamma (96.040, 0.031) (96.040, 0.968)
Discount rate	0.05 (0–0.08) (Attema et al., 2018)		Beta (5.652, 107.395)

AEs, adverse events; ALT, alanine aminotransferase; AST, aspartate aminotransferase; OS, overall survival; PD, progression disease; PFS, progression-free survival; PPES, Palmar-plantar erythrodysaesthesia syndrome.

The baseline utility value for patients in the PFS state was 0.76 for both groups, and 0.68 (Su et al., 2021) for those in the PD state. Negative utility values associated with grade 3/4 AEs were also incorporated into this analysis.

### 2.5 Sensitivity analysis

To assess the robustness of the model, both one-way sensitivity analysis and probabilistic sensitivity analysis were conducted. The one-way sensitivity analysis was used to examine the impact of varying each parameter on the outcomes, with the results graphically depicted in a tornado diagram. In instances where the value range for a parameter was not available, a ±20% variation was assumed for the analysis. The probabilistic sensitivity analysis was performed using Excel, with 1000 Monte Carlo simulations. The findings from this analysis were visualized through a cost-effectiveness acceptability curve, which provides a comprehensive view of the probability that each treatment strategy is cost-effective at various WTP thresholds.

### 2.6 Scenario analysis and subgroup analysis

A scenario analysis with a 10-year time horizon was conducted to evaluate long-term cost-effectiveness. Subgroup analyses were conducted on predefined subgroups from the COSMIC-312 trial. These analyses adjusted the HRs for PFS and OS to explore the influence of these subgroups on the overall results. The calculation method for subgroup analysis is derived from Hoyle et al. (2010), Collett (2014). According to the methods mentioned in these studies, when the OS and PFS of the subgroup and the overall population meet the proportional hazards assumption, the relationship between the survival rates of the two groups and the HR can be derived: the survival rate of the subgroup is equal to the survival rate of the overall population raised to the power of the hazard ratio.

## 3 Results

### 3.1 Base-case results

The results of the baseline analysis were presented in Table 3. The cumulative cost for the sorafenib group in the US was $363,441.38, while for the cabozantinib plus atezolizumab group, it was $539,208.05. In China, the cumulative costs were $30,492.33 for the sorafenib group and $173,674.26 for the cabozantinib plus atezolizumab group. Sorafenib demonstrated a higher number of QALYs with 1.21 compared to 1.15 for the combination group, and a higher number of LYs with 1.70 versus 1.58for the combination group. The combination group incurring a higher cost, and it yielded a lower QALY and a lower LY, resulting in a negative ICER. Consequently, the regimen of cabozantinib in combination with atezolizumab was deemed not cost-effective in both the US and China.

**TABLE 3:** Summary of base-case result and scenario analysis.

Strategy	Cost ($)	QALYs	ICER ($/QALY)	LYs
Base-case result				
US				
Sorafenib	363,441.38	1.21		1.70
Cabozantinib plus Atezolizumab	539,208.05	1.15	−2,731,994.74	1.58
China				
Sorafenib	30,492.33	1.21		1.70
Cabozantinib plus Atezolizumab	173,674.26	1.15	−2,731,994.74	1.58
Scenario analysis result				
US				
Sorafenib	386,702.46	1.27		1.79
Cabozantinib plus Atezolizumab	558,137.28	1.16	−1,621,800.98	1.60
China				
Sorafenib	32,825.14	1.27		1.79
Cabozantinib plus Atezolizumab	180,330.28	1.16	−1,395,422.36	1.60

ICER, incremental cost-effectiveness ratio; LY, life year; QALY, quality-adjusted life year.

### 3.2 Sensitivity analyses

**FIGURE 2:**
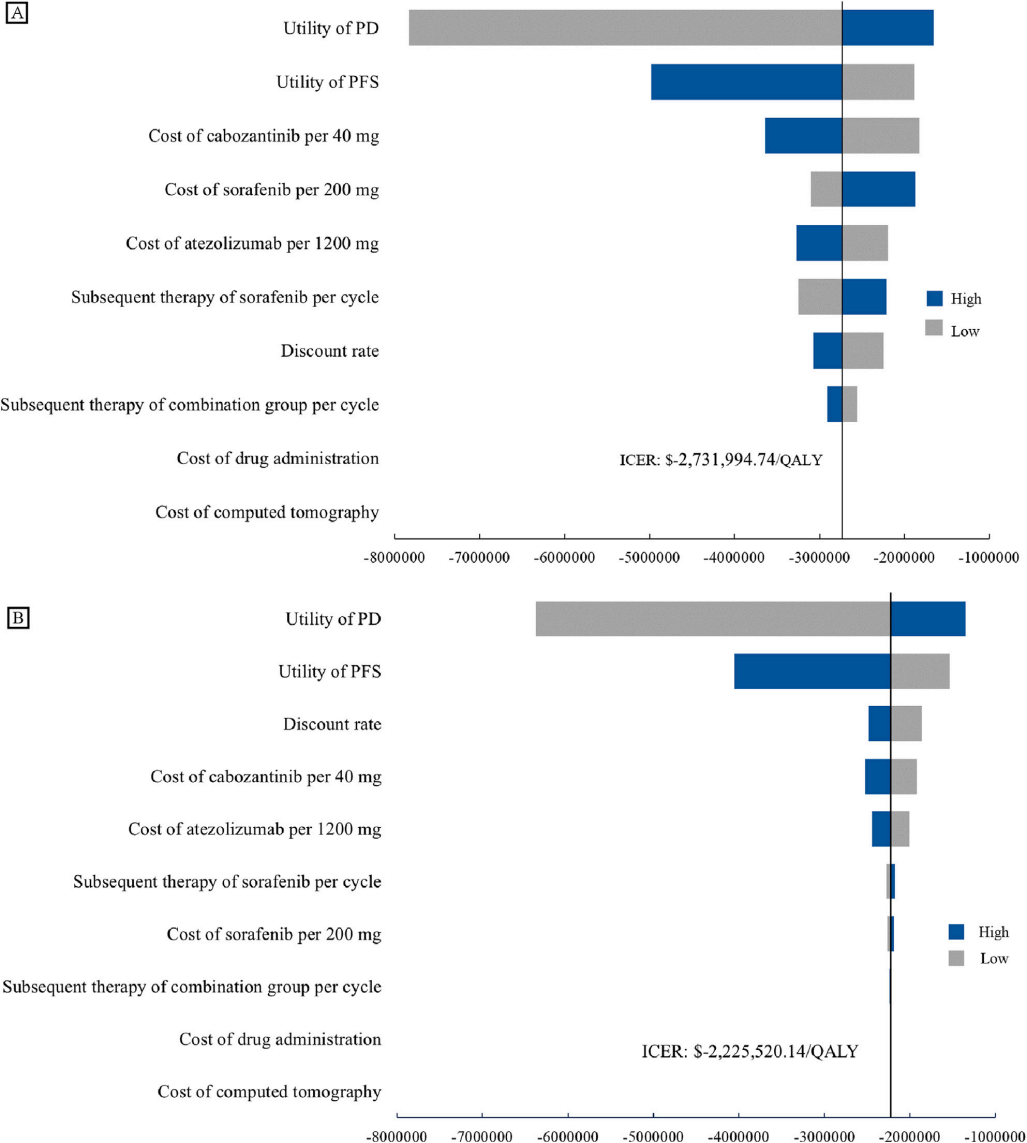
One-way Sensitivity Analyses. Tornado diagrams show the impact on the model from the perspectives of the US **(A)** and China **(B)**. Both tornado diagrams showing one-way sensitivity analyses for each variable in the ±20% range. BSC, best support care; ICER, incremental cost-effectiveness ratio; PD, progression disease; PFS, progression-free survival.

In this model, the tornado diagram from the univariate sensitivity analysis (Figure 2) showed that in the US, the utility values for patients in the PD and PFS states had the greatest impact on the results. These were trailed by the unit cost of cabozantinib, sorafenib and atezolizumab. The costs of subsequent treatment in both the cabozantinib and sorafenib groups exerted a significant impact. In China, utility values for patients with PD and PFS status exert the most significant influence, while discount rates and the unit costs of cabozantinib and atezolizumab also carry substantial weight. Even with ±20% variations in baseline parameter values, conclusions remain consistent with the baseline case analysis, demonstrating the model’s robustness.

**FIGURE 3:**
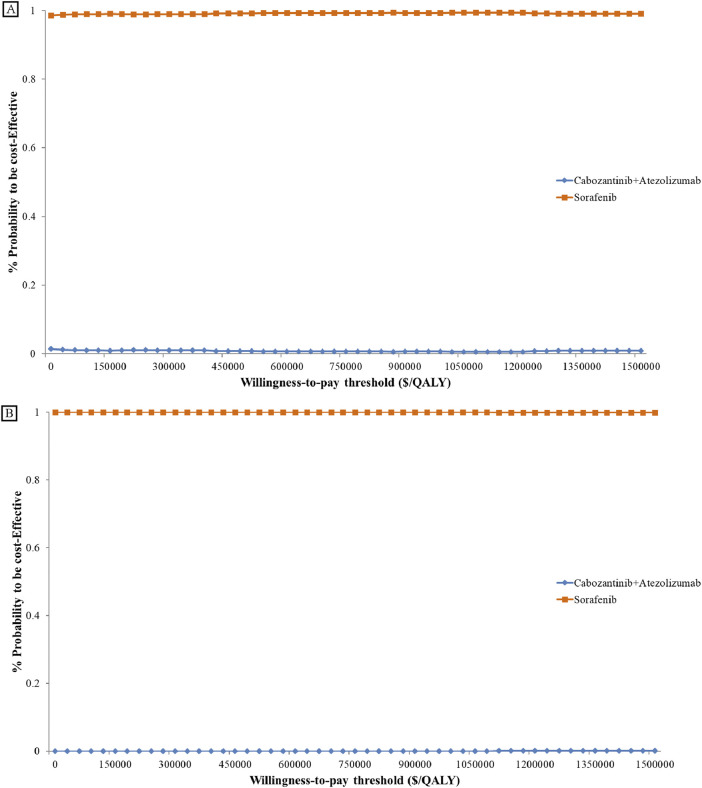
Cost-effectiveness Acceptability Curve. Cost-effectiveness acceptability curves present the probability to be cost-effective of cabozantinib plus atezolizumab compare sorafenib at different WTP thresholds from the US **(A)** and China **(B)**. The y-axis in the graph represents the percentage acceptable and the x-axis represents the willingness-to-pay thresholds QALY, quality-adjusted life-year.

The cost-effectiveness acceptance curve (as shown in Figure 3) indicates that sorafenib demonstrates superior cost-effectiveness compared to the cabozantinib plus atezolizumab regimen for treating advanced hepatocellular carcinoma, regardless of the United States or China.

### 3.3 Scenario analysis and subgroup analyses

Scenario analysis results indicate that, over a 10-year period, the combination group gained 1.19 QALYs, while the sorafenib group gained 1.30 QALYs. Total costs for the combination group were $639,483.16 in the United States and $194,828.76 in China. The total costs for the sorafenib group were $526,145.83 and $47,101.83, respectively. The ICER for cabozantinib plus atezolizumab was -$971,191.65/QALY in the United States and -$1,265,877.51/QALY in China (Table 3).

Subgroup analyses (provided in the Supplementary Table S3) revealed that patients from the Asian region, or with BCLC stage C, hepatitis B virus related liver cancer, or extrahepatic disease or macrovascular invasion, experienced greater survival benefits with cabozantinib plus atezolizumab compared to sorafenib. However, the ICERs in these subgroups still significantly exceeded the WTP threshold. In other subgroups, sorafenib remained dominant, offering more QALYs at a lower cost.

## 4 Discussion

This study constructed a partitioned survival model based on data from the COSMIC-312 trial and relevant literature to evaluate the cost-effectiveness of cabozantinib plus atezolizumab versus sorafenib as first-line treatments for advanced hepatocellular carcinoma from the perspectives of the US payers and the Chinese healthcare system. The results showed that the combination therapy yielded fewer QALYs and LYs over a five-year time horizon compared to sorafenib. Moreover, the combination therapy incurred significantly higher costs than sorafenib in both the US and China. Consequently, the combination of cabozantinib and atezolizumab is not likely to be considered cost-effective as a first-line treatment option in either country. Although subgroup analyses suggested that certain subgroups derived more significant survival benefits from the combination therapy, the substantial costs of the drugs made the combination therapy an unlikely cost-effective choice.

Due to the high cost of healthcare in the United States, the total cost of the two treatment strategies and their difference are proportionally smaller. In China, although the total cost of the combination therapy group is nearly four times that of the sorafenib group, the relative cost difference is not significantly different from the values observed in the United States. Therefore, the ICER is similar in both countries. Regarding the WTP threshold, the threshold we assumed for the United States is approximately four times that of China, which is related to the development levels and national consumption capacity of the two countries. Although the threshold setting is rough, it can serve as a reasonable assumption when analyzing whether an investment is justified (Neumann et al., 2014).

Previous trials in advanced HCC have reported clinical trial results for ICIs or TKIs as monotherapy, or combination strategies involving multiple ICIs (Finn et al., 2020; Llovet et al., 2021; Abou-Alfa et al., 2018; Yau et al., 2022). The combination of cabozantinib and atezolizumab represented the first Phase III randomized controlled trial to assess the synergistic effect of a TKI and an ICI. While this combination demonstrated some potential in delaying disease progression, clinical studies have shown that this benefit in PFS did not translate into OS benefit. In fact, the overall survival of the combination therapy group showed a declining trend compared to the standard first-line treatment, sorafenib. In our study, leveraging the fitted survival data and the extrapolation of survival curves, sorafenib even demonstrated superior survival benefits after disease progression compared to the combination therapy (0.67 QALYs vs. 0.46 QALYs). The reasons for this finding are currently unknown, but it is speculated that the higher toxicity associated with the combination therapy in clinical trials may have led to dose reductions or treatment interruptions, ultimately affecting overall survival. Additionally, the rate of accepted subsequent treatment in the sorafenib group was higher than that in the combination therapy group, and this rate was influenced by multiple factors. Overall, the results for overall survival may be underestimated.

Systemic therapies for unresectable advanced HCC have seen significant advancements in recent years. The success of atezolizumab plus bevacizumab and durvalumab plus tremelimumab has marked a breakthrough in systemic treatment, transcending the traditional standard of care that relied solely on TKIs. Immunotherapy is poised to become a more popular option. As clinical trial results for these new therapies have been published, accompanying pharmacoeconomic analyses have been undertaken. However, almost all studies have indicated that, in comparison to sorafenib, combination immunotherapy is unlikely to be cost-effective Wen et al. (2021), Su et al. (2021). Evaluated the cost-effectiveness of atezolizumab plus bevacizumab versus sorafenib as first-line treatments for advanced HCC patients from the perspectives of the Chinese healthcare system and the US payers, respectively. Both studies found that even though the combination of atezolizumab and bevacizumab significantly improved PFS and OS, its high cost made the regimen unaffordable, and the ICER of the combination therapy exceeded the WTP threshold. In our study, univariate sensitivity analyses demonstrated that drug pricing was a substantial determinant of cost-effectiveness, suggesting that reductions in drug prices could render the combination therapy more cost-effective. When the total prices of cabozantinib and atezolizumab were reduced to 59.8% and 13.1% of their baseline values in the US and China, respectively, the combination therapy became more favorable. This means that the cabozantinib plus atezolizumab strategy achieves a slightly lower QALY at a lower cost, making it a reasonable choice compared to sorafenib.

Our study had several limitations. Firstly, the majority of the cost data in this study were obtained from published literature rather than from real-world data, which may introduce some bias. However, univariate sensitivity analysis showed that these cost data had a relatively small impact on the results. Secondly, utility values, as key parameters in pharmacoeconomic evaluations, are influenced by regional, ethnic, and cultural differences. The utility values utilized in this study were extracted from a clinical trial that spanned 178 centers across 32 countries, encompassing regions such as Asia and Europe. Additionally, univariate sensitivity analysis showed that utility values for patients in the PD and PFS states had the greatest impact on the results, potentially leading to biased outcomes. Thirdly, to streamline the model, only severe adverse events (grade 3/4) were included in this study, which might introduce some bias. However, sensitivity analysis showed that these parameters had a limited impact on the results. Furthermore, the follow-up time in the COSMIC-312 trial was relatively short, and the extrapolation of long-term efficacy through survival curve fitting may not accurately reflect the actual long-term effects in real-world settings.

## 5 Conclusion

In conclusion, from the perspectives of the US payers and the Chinese healthcare system, cabozantinib plus atezolizumab as first-line therapy for HCC is unlikely to be cost-effective compared to sorafenib, despite the potential progression-free survival benefits.

## Data Availability

The original contributions presented in the study are included in the article/Supplementary Material, further inquiries can be directed to the corresponding authors.
