# Correction: Cost-effectiveness analysis of cabozantinib plus atezolizumab for advanced hepatocellular carcinoma

**DOI:** 10.3389/fphar.2025.1735585

**Published:** 2025-11-13

**Authors:** Yan Zhu, Cheng He, Shi-Chao Cao, Hui-Jun Li, Ru-Xue Cai, Zhao-Jun Bai, Tong-Xia Xia

**Affiliations:** 1 Department of Pharmacy, Affiliated Hospital of Zunyi Medical University, Zunyi, Guizhou, China; 2 Department of Pharmacy, Zunyi Bozhou District People’s Hospital, Zunyi, Guizhou, China; 3 Sinopharm Group Tongjitang (Guizhou) Pharmaceutical Co., Ltd., Economic and Technological Development District, Guiyang, Guizhou, China; 4 Guangxi Shenli Pharmaceutical Co., Ltd., Yulin, Guangxi, China; 5 School of Nursing, Zunyi Medical University, Zunyi, Guizhou, China

**Keywords:** cost-effectiveness analysis, advanced hepatocellular carcinoma, cabozantinib, atezolizumab, sorafenib

**Affiliation 1** Department of Pharmacy, Affiliated Hospital of Zunyi Medical University, Zunyi, Guizhou, China was omitted for author Tong-Xia Xia. This affiliation has now been added for author Tong-Xia Xia.

The original article has been updated.

