# Supplementary material for: Cost-effectiveness analysis of cabozantinib plus atezolizumab for advanced hepatocellular carcinoma
Source: Front Pharmacol. 2025 Oct 9;16:1556304. doi: 10.3389/fphar.2025.1556304 (PMC12545015; doi:10.3389/fphar.2025.1556304)

***Supplementary Materials***

**Table S1.** CHEERS 2022 Checklist

**Table S2.** The Akaike information criterion (AIC) measures of goodness of fit for the comparison of survival models

**Table S3.** Results of subgroup analyses

**Figure S1.** Curve fitting to reconstructed survival curves

**Table S1.** CHEERS 2022 Checklist

| **Topic** | **No.** | **Item** | **Location where item is reported** |
| --- | --- | --- | --- |
| **Title** |  |  |  |
|  | 1 | Identify the study as an economic evaluation and specify the interventions being compared. | Title |
| **Abstract** |  |  |  |
|  | 2 | Provide a structured summary that highlights context, key methods, results, and alternative analyses. | Abstract |
| **Introduction** |  |  |  |
| **Background and objectives** | 3 | Give the context for the study, the study question, and its practical relevance for decision making in policy or practice. | 1 Introduction |
| **Methods** |  |  |  |
| **Health economic analysis plan** | 4 | Indicate whether a health economic analysis plan was developed and where available. | Not reported |
| **Study population** | 5 | Describe characteristics of the study population (such as age range, demographics, socioeconomic, or clinical characteristics). | 2.1 Population and interventions, 1^st^ sentence |
| **Setting and location** | 6 | Provide relevant contextual information that may influence findings. | 2.2 Model overview |
| **Comparators** | 7 | Describe the interventions or strategies being compared and why chosen. | 2.1 Population and interventions, 2^nd^ sentence |
| **Perspective** | 8 | State the perspective(s) adopted by the study and why chosen. | 2.2 Model overview, 6^th^ sentence |
| **Time horizon** | 9 | State the time horizon for the study and why appropriate. | 2.2 Model overview, 4^th^ sentence |
| **Discount rate** | 10 | Report the discount rate(s) and reason chosen. | 2.2 Model overview, 10 sentence |
| **Selection of outcomes** | 11 | Describe what outcomes were used as the measure(s) of benefit(s) and harm(s). | 2.2 Model overview, 7^th^ sentence |
| **Measurement of outcomes** | 12 | Describe how outcomes used to capture benefit(s) and harm(s) were measured. | 2.2 Model overview, 7^th^ sentence |
| **Valuation of outcomes** | 13 | Describe the population and methods used to measure and value outcomes. | 2.4 Cost and Utility, 3^rd^ paragraph |
| **Measurement and valuation of resources and costs** | 14 | Describe how costs were valued. | 2.4 Cost and Utility, 2^nd^ paragraph |
| **Currency, price date, and conversion** | 15 | Report the dates of the estimated resource quantities and unit costs, plus the currency and year of conversion. | 2.4 Cost and Utility, 1^st^ paragraph |
| **Rationale and description of model** | 16 | If modelling is used, describe in detail and why used. Report if the model is publicly available and where it can be accessed. | 2.2 Model overview |
| **Analytics and assumptions** | 17 | Describe any methods for analysing or statistically transforming data, any extrapolation methods, and approaches for validating any model used. | 2.2 Model overview, 2.3 Survival estimate |
| **Characterising heterogeneity** | 18 | Describe any methods used for estimating how the results of the study vary for subgroups. | 2.6 Scenario analysis and subgroup analysis |
| **Characterising distributional effects** | 19 | Describe how impacts are distributed across different individuals or adjustments made to reflect priority populations. | Not reported |
| **Characterising uncertainty** | 20 | Describe methods to characterise any sources of uncertainty in the analysis. | 2.5 Sensitivity analysis |
| **Approach to engagement with patients and others affected by the study** | 21 | Describe any approaches to engage patients or service recipients, the general public, communities, or stakeholders (such as clinicians or payers) in the design of the study. | Not reported |
| **Results** |  |  |  |
| **Study parameters** | 22 | Report all analytic inputs (such as values, ranges, references) including uncertainty or distributional assumptions. | 2.3 Survival estimate, 2.4 Cost and Utility, Table 1, Table 2 |
| **Summary of main results** | 23 | Report the mean values for the main categories of costs and outcomes of interest and summarise them in the most appropriate overall measure. | 3.1 Base-case results, Table 3 |
| **Effect of uncertainty** | 24 | Describe how uncertainty about analytic judgments, inputs, or projections affect findings. Report the effect of choice of discount rate and time horizon, if applicable. | 3.2 Sensitivity analyses, Figure 2, Figure 3 |
| **Effect of engagement with patients and others affected by the study** | 25 | Report on any difference patient/service recipient, general public, community, or stakeholder involvement made to the approach or findings of the study | Not reported |
| **Discussion** |  |  |  |
| **Study findings, limitations, generalisability, and current knowledge** | 26 | Report key findings, limitations, ethical or equity considerations not captured, and how these could affect patients, policy, or practice. | 4 Discussion |
| **Other relevant information** |  |  |  |
| **Source of funding** | 27 | Describe how the study was funded and any role of the funder in the identification, design, conduct, and reporting of the analysis | Funding |
| **Conflicts of interest** | 28 | Report authors conflicts of interest according to journal or International Committee of Medical Journal Editors requirements. | Conflict of Interest |

*From:* Husereau D, Drummond M, Augustovski F, et al. Consolidated Health Economic Evaluation Reporting Standards 2022 (CHEERS 2022) Explanation and Elaboration: A Report of the ISPOR CHEERS II Good Practices Task Force. Value Health 2022;25. <doi:10.1016/j.jval.2021.10.008>

**Table S2.** The Akaike information criterion (AIC) and Bayesian information criterion (BIC) measures of goodness of fit for the comparison of survival models

| Distribution | | OS | | PFS | |
| --- | --- | --- | --- | --- | --- |
|  | AIC | | BIC | AIC | BIC |
| Weibull | 1043.73 | | 1051.867 | 1143.877 | 1152.014 |
| Exponential | 1064.883 | | 1068.952 | 1145.609 | 1149.678 |
| Gompertz | 1052.095 | | 1060.232 | 1146.621 | 1154.758 |
| Loglogistic | 1044.884 | | 1053.021 | 1114.361 | 1122.498 |
| Lognormal | 1049.097 | | 1057.234 | 1102.114 | 1102.114 |

1. Cabozantinib + atezolizumab arm.
2. Sorafenib arm.

| Distribution | | OS | | PFS | |
| --- | --- | --- | --- | --- | --- |
|  | AIC | | BIC | AIC | BIC |
| Weibull | 544.582 | | 551.3418 | 546.1808 | 552.9405 |
| Exponential | 543.8652 | | 547.2451 | 544.1868 | 547.5667 |
| Gompertz | 545.7045 | | 552.4643 | 537.4043 | 544.1641 |
| Loglogistic | 537.9052 | | 544.665 | 517.6785 | 524.4383 |
| Lognormal | 532.8219 | | 539.5817 | 509.7038 | 516.4636 |

**Table S3.** Results of subgroup analyses

|  | **Sample size** | | **Reported HRs** | | **ICER ($/QALY)** | |  |
| --- | --- | --- | --- | --- | --- | --- | --- |
| **Subgroups** | **Cabozantinib plus atezolizumab** | **Sorafenib** | **HR for PFS (95% CI)** | **HR for OS (95%CI)** | **US** | **China** |  |
| **Sex** |  |  |  |  |  |  |  |
| Female | 72 | 31 | 1·18 (0·67–2·06) | 1.23(0.69-2.22) | 1,777,517.26 | 1,058,714.16 |  |
| Male | 360 | 186 | 0·71 (0·57–0·89) | 0·98 (0·78–1·24) | -2,132,500.05 | -2,568,632.87 |  |
| **Region** |  |  |  |  |  |  |  |
| Asia | 120 | 63 | 0·71 (0·57–0·89) | 0·77 (0·50–1·18) | -448,856.58 | -507,811.65 |  |
| Other | 312 | 154 | 0·89 (0·69–1·13) | 1·12 (0·87–1·44) | 3,930,371.86 | 2,662,106.93 |  |
| **ECOG performance status** |  |  |  |  |  |  |  |
| 0 | 267 | 143 | 0·74 (0·57–0·95) | 1·16 (0·87–1·54) | 2,912,903.49 | 2,039,395.28 |  |
| 1 | 154 | 74 | 0·85 (0·60–1·20) | 0·79 (0·57–1·10) | -592,278.10 | -582,784.85 |  |
| **BCLC stage** |  |  |  |  |  |  |  |
| Category B | 140 | 72 | 1·03 (0·72–1·49) | 1·25 (0·85–1·84) | 1,668,956.65 | 1,009,701.96 |  |
| Category C | 292 | 145 | 0·66 (0·52–0·85) | 0·90 (0·70–1·17) | -1,033,330.99 | -9,882,838.20 |  |
| **Baseline alpha-fetoprotein, ng/mL** |  |  |  |  |  |  |  |
| <400 | 269 | 152 | 0·85 (0·66–1·10) | 1·22 (0·92–1·62) | 1,922,806.91 | 1,239,406.35 |  |
| ≥400 | 163 | 65 | 0·54 (0·38–0·77) | 0·60 (0·43–0·84) | -83,779.51 | -259,243.10 |  |
| **Disease aetiology** |  |  |  |  |  |  |  |
| HBV (with or without HCV) | 127 | 64 | 0·52 (0·37–0·75) | 0·63 (0·42–0·95 | -103,652.09 | -282,415.81 |  |
| HCV (without HBV) | 136 | 67 | 0·78 (0·53–1·13) | 1·13 (0·78–1·63) | 3,821,480.66 | 2,680,657.89 |  |
| Non-viral | 169 | 86 | 0·99 (0·71–1·39) | 1·28 (0·90–1·83) | 1,517,601.87 | 2,182,245.75 |  |
| **Non-viral aetiology: alcoholism** |  |  |  |  |  |  |  |
| Yes | 59 | 33 | 0·89 (0·51–1·55) | 1·26 (0·71–2·22) | 1,526,224.73 | 1,015,154.56 |  |
| No | 109 | 52 | 1·03 (0·67–1·57) | 1·27 (0·80–2·02) | 1,561,768.01 | 935,466.60 |  |
| **Non-viral aetiology: NASH or NAFLD** | |  |  |  |  |  |  |
| Yes | 48 | 22 | 1·32 (0·67–2·63) | 1·72 (0·83–3·55) | 1,545,110.43 | 407,638.85 |  |
| No | 113 | 59 | 1·00 (0·67–1·49) | 1·09 (0·72–1·65) | 939,109.72 | 507,138.80 |  |
| **Non-viral aetiology: other** |  |  |  |  |  |  |  |
| Yes | 76 | 40 | 0·90 (0·56–1·44) | 1·04 (0·63–1·71) | -49,337,467.52 | -35,373,362.99 |  |
| No | 93 | 46 | 1·10 (0·68–1·79) | 1·55 (0·93–2·59) | 943,336.63 | 28,737,565.00 |  |
| **Child–Pugh classification** |  |  |  |  |  |  |  |
| A5 | 338 | 178 | 0·79 (0·63–0·99) | 1·02 (0·79–1·30) | -6,933,268.10 | -5,288,293.71 |  |
| A6 | 78 | 20 | 0·96 (0·52–1·79) | 0·87 (0·49–1·53) | -1,131,560.43 | -943,893.40 |  |
| **ALBI grade** |  |  |  |  |  |  |  |
| Grade 1 | 249 | 123 | 0·87 (0·65–1·16) | 1·03 (0·76–1·41) | -14,372,419.79 | -10,502,687.42 |  |
| Grade 2 | 182 | 89 | 0·65 (0·48–0·88) | 0·97 (0·71–1·31) | -1,983,108.91 | -1,741,450.04 |  |
| **Cirrhosis** |  |  |  |  |  |  |  |
| Yes | 291 | 142 | 0·74 (0·58–0·96) | 0·95 (0·73–1·23) | -1,877,934.24 | -1,580,208.00 |  |
| No | 139 | 75 | 0·83 (0·59–1·19) | 1·17 (0·79–1·74) | 2,568,119.15 | 1,718,168.15 |  |
| **Macrovascular invasion** |  |  |  |  |  |  |  |
| Yes | 136 | 61 | 0·51 (0·36–0·74) | 0·77 (0·53–1·12) | -298,635.48 | -459,062.42 |  |
| No | 296 | 156 | 0·90 (0·70–1·15) | 1·11 (0·85–1·44) | 4,441,328.93 | 3,019,476.38 |  |
| **Extrahepatic disease** |  |  |  |  |  |  |  |
| Yes | 232 | 123 | 0·77 (0·59–1·00) | 0·92 (0·70–1·22) | -1,448,353.49 | -1,239,255.66 |  |
| No | 200 | 94 | 0·82 (0·59–1·14) | 1·14 (0·81–1·59) | 3,316,760.05 | 2,271,984.66 |  |
| **Extrahepatic disease or macrovascular invasion** | |  |  |  |  |  |  |
| Yes | 298 | 148 | 0·65 (0·51–0·83) | 0·87 (0·68–1·13) | -806,495.01 | -809,203.38 |  |
| No | 134 | 69 | 1·11 (0·75–1·65) | 1·34 (0·89–2·02) | 1,297,658.30 | 742,142.45 |  |

Abbreviation: ALBI, albumin-bilirubin. BCLC, Barcelona Clinic Liver Cancer. ECOG, Eastern Cooperative Oncology Group. HBV, hepatitis B virus. HCV, hepatitis C virus. HR, hazard ratio. NAFLD, non-alcoholic fatty liver disease. NASH, non-alcoholic steatohepatitis

**Figure S1.** Curve fitting to reconstructed survival curves

1. Model fitting of the observed survival curves in cabozantinib + atezolizumab arm.


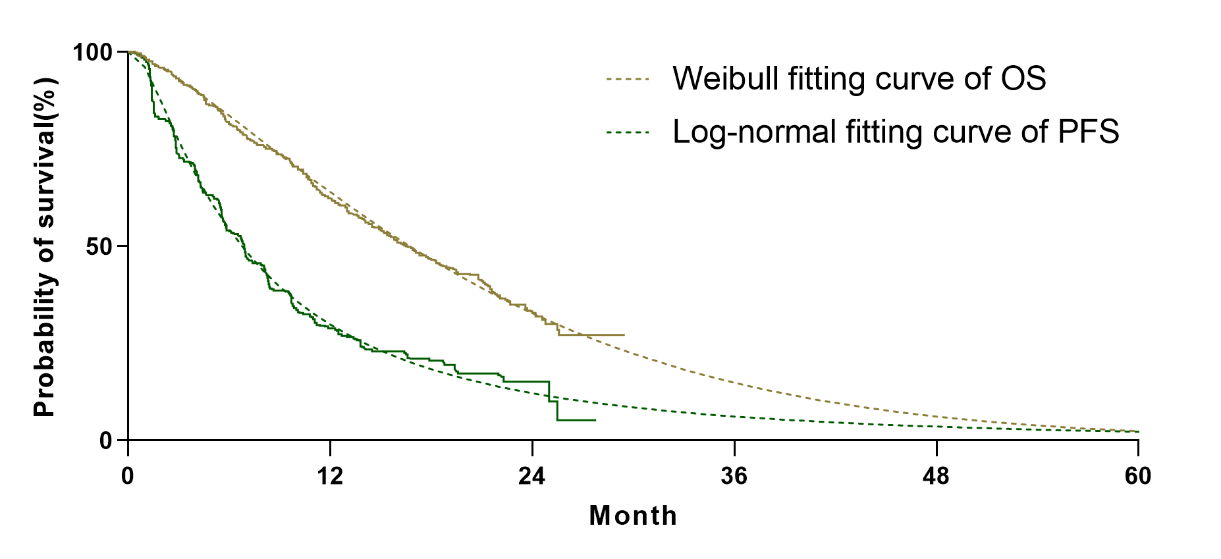


1. Model fitting of the observed survival curves in sorafenib arm.


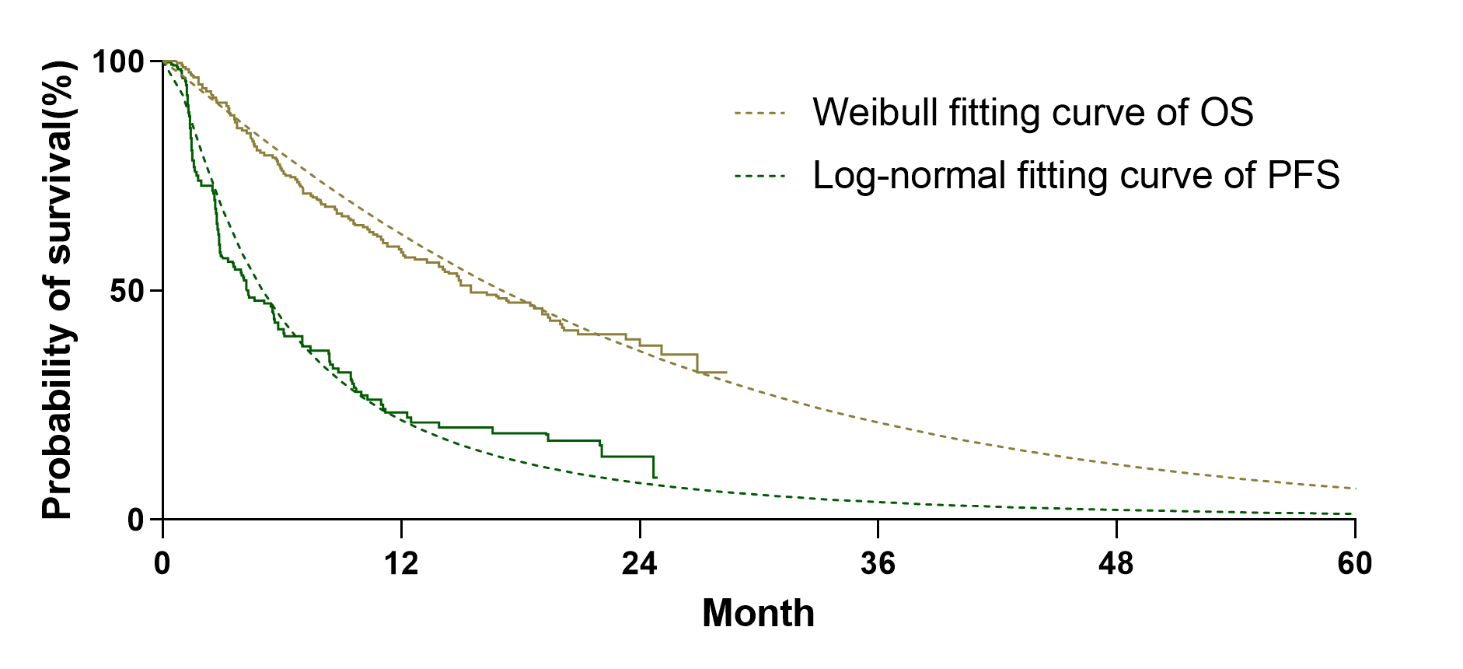

Supplement: Supplementary file 1 [file Supplementaryfile1.docx]
